# Oscillatory mechanisms of selective integration during decision making

**DOI:** 10.1186/1471-2202-12-S1-O16

**Published:** 2011-07-18

**Authors:** Angela CE Onslow, Matthew W Jones, Rafal Bogacz

**Affiliations:** 1 Bristol Centre for Complexity Sciences, University of Bristol, Bristol, BS8 1TR, UK; 2 School of Physiology & Pharmacology, University of Bristol, Bristol, BS8 1TD, UK; 3 Department of Computer Science, University of Bristol, Bristol, BS8 1UB, UK

## 

Coordinated, rhythmic neuronal activity is proposed to allow selective routing of information by downstream targets which can filter a rhythmic input from noisy, asynchronous inputs. This mechanism was recently implemented in a model by Akam and Kullman [1]. Our work extends their concept to incorporate a neural mechanism for integrating the routed information, intended to model decision making. In our model, oscillatory input signals encode information relevant to the decision making task which must be evaluated in order to make a correct decision. The model is based on hippocampal-prefrontal interactions during a spatial working memory task. In this context the oscillatory signal represents the working memory item being retrieved and is assumed to be generated through an interactive process between the hippocampus and prefrontal cortex. This information is then integrated by prefrontal neurons which will initiate a decision once an integration threshold is reached. An oscillatory (as opposed to asynchronous) signal allows the relevant information to be filtered from other simultaneous activity and attended to by the cortex.

Two variations of the model are explored: one which combines routing and integration in a single subunit of the model (equivalent to a single processing step); the other utilizes two separate subunits, one for routing and one for integration. The model components represent excitatory pyramidal cells and inhibitory interneurons in prefrontal cortex. The two models make different experimental predictions which are evaluated through comparison with data recorded in rats during a task which requires spatial working memory (specifically, the memory of the last turn made) in order to make a correct decision [2].
